# α-Melanocyte Stimulating Hormone Treatment in Pigs Does Not Improve Early Graft Function in Kidney Transplants from Brain Dead Donors

**DOI:** 10.1371/journal.pone.0094609

**Published:** 2014-04-11

**Authors:** Willem G. van Rijt, Niels Secher, Anna K. Keller, Ulla Møldrup, Yahor Chynau, Rutger J. Ploeg, Harry van Goor, Rikke Nørregaard, Henrik Birn, Jørgen Frøkiaer, Søren Nielsen, Henri G. D. Leuvenink, Bente Jespersen

**Affiliations:** 1 Department of Surgery, University Medical Center Groningen, Groningen, The Netherlands; 2 Department of Anesthesiology, Aarhus University Hospital, Aarhus, Denmark; 3 Department of Urology, Aarhus University Hospital, Aarhus, Denmark; 4 Nuffield Department of Surgical Sciences, University of Oxford, Oxford, United Kingdom; 5 Department of Pathology and Medical Biology, University Medical Center Groningen, Groningen, The Netherlands; 6 Department of Clinical Medicine, Aarhus University Hospital, Aarhus, Denmark; 7 Department of Renal Medicine, Aarhus University Hospital, Aarhus, Denmark; 8 The Water and Salt Research Center, Department of Biomedicine, Aarhus University, Aarhus, Denmark; 9 The Water and Salt Research Center, Department of Clinical Medicine, Aarhus University, Aarhus, Denmark; UNIFESP Federal University of São Paulo, Brazil

## Abstract

Delayed graft function and primary non-function are serious complications following transplantation of kidneys derived from deceased brain dead (DBD) donors. α-melanocyte stimulating hormone (α-MSH) is a pleiotropic neuropeptide and its renoprotective effects have been demonstrated in models of acute kidney injury. We hypothesized that α-MSH treatment of the recipient improves early graft function and reduces inflammation following DBD kidney transplantation. Eight Danish landrace pigs served as DBD donors. After four hours of brain death both kidneys were removed and stored for 18 hours at 4°C in Custodiol preservation solution. Sixteen recipients were randomized in a paired design into two treatment groups, transplanted simultaneously. α-MSH or a vehicle was administered at start of surgery, during reperfusion and two hours post-reperfusion. The recipients were observed for ten hours following reperfusion. Blood, urine and kidney tissue samples were collected during and at the end of follow-up. α-MSH treatment reduced urine flow and impaired recovery of glomerular filtration rate (GFR) compared to controls. After each dose of α-MSH, a trend towards reduced mean arterial blood pressure and increased heart rate was observed. α-MSH did not affect expression of inflammatory markers. Surprisingly, α-MSH impaired recovery of renal function in the first ten hours following DBD kidney transplantation possibly due to hemodynamic changes. Thus, in a porcine experimental model α-MSH did not reduce renal inflammation and did not improve short-term graft function following DBD kidney transplantation.

## Introduction

Kidneys of deceased brain dead (DBD) donors are the main source of kidneys for transplantation world-wide. In 2012, 64% of all renal transplants in Europe were from DBD donors [1]. Despite significant advances in recipient management, delayed graft function (DGF) and primary non-function (PNF) remain as serious complications of DBD donor renal transplantation occurring in 18–28% and 2–4% of recipients respectively [2]–[4]. DGF is associated with additional burden to the patient and with increased rejection risk, reduced graft survival and higher costs associated with extended hospital admission. Thus, outcome from DBD donation remains inferior to living donation. Preventing injury to the DBD kidney allograft may improve short-term kidney function and also influence longer term graft survival.

Brain death induces a systemic, inflammatory state. This is caused by hemodynamic changes and neuronal injury. Cytokines such as IL-6 and MCP-1 mediate leukocyte infiltration that occurs alongside systemic complement system activation [5]–[8]. This systemic response results in inflammatory activation of the donor end organ, which is increased by hemodynamic instability [8]. Overall, brain death results in injured organs even prior to organ retrieval. This injury is worsened by the additive effects of preservation and ischemia/reperfusion (I/R) injury [9]. As the major part of I/R injury arises during the reperfusion phase, renoprotective treatment of recipients is an attractive therapeutic option.

α-melanocyte stimulating hormone is a pleiotropic neuropeptide with renoprotective capacities demonstrated in several models of acute kidney injury including cyclosporine induced nephrotoxicity [10], ureteral obstruction [11] and I/R injury [12]–[17]. In renal I/R models α-MSH administered up to six hours post-reperfusion improved renal function and resulted in reduced acute tubular necrosis and neutrophil influx [12]. The protective effect is not fully dependent on inhibition of neutrophil activation, as α-MSH was still protective in renal I/R in ICAM-1 knock-out mice [13]. In addition, α-MSH prevented the down-regulation of aquaporins and sodium transporters involved in tubular reabsorbtion of water following acute kidney injury [11], [16].

As DBD donor kidney allografts are affected by the brain death process and I/R injury, α-MSH treatment of the recipient may post-condition kidneys to improve short-term renal function following transplantation and reduce the incidence of DGF and PNF. We therefore hypothesized that α-MSH treatment of the recipient protects against renal inflammation and I/R injury in a porcine model of DBD kidney transplantation leading to improved early graft function.

## Materials and Methods

### Animals and ethics statement

Twenty-four Danish Landrace pigs (50–65 kg.) were used. The pigs were fasted overnight before surgery with free access to water. The animal experiments were performed in strict accordance with international and Danish guidelines of animal research. The study protocol was approved by the Danish Animal Experiments Inspectorate and included moving, sedation and surgery of the animals (permit number: 2012-15-2934-00122). All surgery was performed under anesthesia and all efforts were made to minimize suffering. Samples size of eight animals per treatment group was calculated based on a 2-sided α of 0.05, a power of 0.9 and an effect size of 1.92.

### Study design

**Figure 1:**
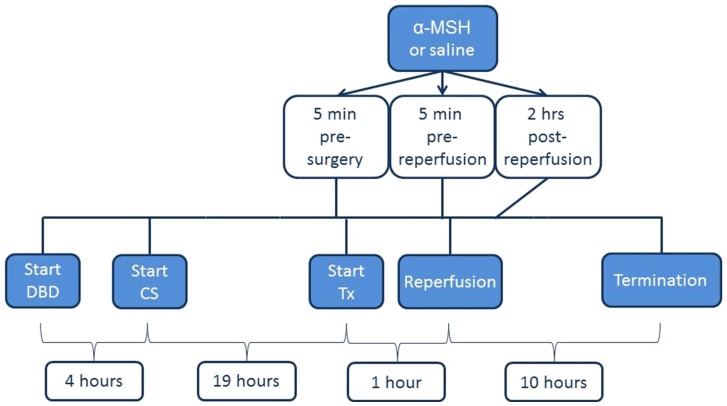
Study design.

To test our hypothesis, we used a randomized, paired design. Eight pigs were used as DBD donors and both kidneys were transplanted. After four hours of brain death both kidneys were removed and cold storage lasted nineteen hours. Donor kidneys derived from the same donor were transplanted simultaneously to one α-MSH- and one vehicle treated recipient. The follow-up was ten hours following reperfusion. The study was investigator-blinded and recipients were randomized in a paired design into two treatment groups. Surgeons and right- and left kidneys were also randomized into the two treatment groups. α-MSH (Bachem, Bubendorf, Switzerland) was dosed at 200 µg/kg and saline (0,9%) served as vehicle treatment. 200 µg/kg was chosen based on its protective effect against renal I/R injury as shown by Gong et al. and Simmons et al. [16], [17]. Both treatments (0.2 ml/kg) were infused over ten minutes. Administration was started five minutes prior to start of abdominal surgery, five minutes prior to reperfusion and two hours post-reperfusion. The study design is shown in figure 1.

### Anesthesia and monitoring

Before transport to the animal operation facility, pigs were sedated by intramuscular injections of azaperone (0.1 ml/kg) and midazolam (0.5 mg/kg). At arrival midazolam (0.5 mg/kg), ketamine (5 mg/kg) and atropine (0.01 mg/kg) was given to prolong sedation. Prior to intubation midazolam (0.5 mg/kg) and ketamine (5 mg/kg) was administered intravenously. After intubation continuous anesthesia was maintained using propofol (8 mg/kg/hr) and fentanyl (25 µg/kg/hr). Animals were ventilated with 40% oxygen and a tidal volume of 10 ml/kg. Expiratory CO2 was controlled between 4.5- and 5.5 kPA by adjusting respiratory rate. Ringer acetate was infused continuously (donors: 10 ml/kg/hr; recipients: 15 ml/kg/hr). The carotid artery and jugular vein were catheterized. Blood pressure was monitored via a pressure catheter in the carotid artery. Mean arterial pressure (MAP) was maintained above 60 mmHg. If MAP decreased below 60 mmHg a bolus infusion of one liter Ringer's acetate was given and if not sufficient to maintain MAP above 60 mmHg an intravenous bolus of adrenalin (0.05 mg) was administered. Cefuroxime (750 mg) was administered as antibiotic treatment before start of surgery and repeated after six hours. A bolus of 20 ml 50% glucose was administered if the blood glucose level dropped below 4.0 mmol/l.

### Deceased brain dead kidney donation

For brain dead induction two holes were drilled in the cranium. Intracranial pressure was measured through the first hole and via the secondary hole a 22Fr 60cc Foley urine catheter was inserted in the epidural space. Brain death was induced by inflating the balloon of the 22Fr 60cc Foley urine catheter with saline at a rate of 1ml/min. The exact time of brain death was determined when intracranial pressure was higher than the mean arterial pressure [18], [19]. At this point ten ml extra saline was infused in the intracranial catheter (1 ml/min) to ensure no circulation of the brain. To prevent muscle cramps rocuronium (130 mg) was administered intravenously. Continuous propofol administration was stopped after declaration of brain death.

After four hours of brain death, donor surgery was started by midline laparotomy. Both kidneys were dissected. Prior to removal of the kidneys heparin (20000 IU) was given and kidneys were flushed in situ with one liter of 4°C Custodiol. After donation kidneys were preserved at 4°C for 19 hours.

### Transplantation

The transplantation procedure in recipients was started by a midline incision and both kidneys were approached retroperitoneally. Right- and left nephrectomy was performed and donor kidneys were transplanted by end-to-end anastomoses to the left renal artery and vein. During surgery the organs were cooled with frozen glucose. The ureter was catheterized with a 10Fr feeding tube. Fifteen minutes post-reperfusion the abdomen was closed. After ten hours of follow-up the recipients were sacrificed using pentobarbital (80 mg/kg) while still under anesthesia.

### Samples

Blood and urine samples were taken via a carotid artery catheter and catheterized ureters, respectively. Blood and urine samples of the recipient were obtained at the moment of reperfusion, each 30 minutes in the first 2 hours and then hourly till end of follow-up.

Blood and urine samples were stored at −80°C. Cortical and medullary samples of the kidney were snap frozen in liquid N_2_ and stored at −80°C for qRT-PCR. For immunohistochemistry kidney tissue samples were fixated in 4% formalin and subsequently embedded in paraffin.

### Glomerular filtration rate

The primary endpoint of the study was renal function defined as GFR measuring ^51^Cr-EDTA clearance. An intravenous bolus of ^51^Cr-EDTA (2.6 mBq) was administered followed by continuous infusion of ^51^Cr-EDTA (1.3 mBq/hour). The activity in blood and urine samples was counted using a gamma ray detector (Cobra II, Packard, Meriden, CT). Values were corrected for decay. The GFR was calculated using the following formula: GFR  =  (urinary ^51^Cr-EDTA (CPM/ml) * urine flow (ml/min))/plasma ^51^Cr-EDTA (CPM/ml).

### Quantitative real-time reverse transcription polymerase chain reaction (qRT-PCR)

RNA was extracted from snap frozen tissue using Trizol reagent according to the manufacturer's instructions (Invitrogen, Breda, the Netherlands). Total RNA was treated with DNAse I to remove genomic DNA contamination (Invitrogen, Breda, the Netherlands). The integrity of total RNA was analyzed by gel electrophoresis. cDNA was synthesized from 1-µg total RNA using M-MLV (Moloney murine leukaemia virus) Reverse Transcriptase and oligo-dT primers (Invitrogen, Breda, The Netherlands).

Primer sets were designed using Primer Express 2.0 software (Applied Biosystems, Foster City, CA). Amplification and detection were performed with the ABI Prism 7900-HT Sequence Detection System (Applied Biosystems) using emission from SYBR green master mix (Applied Biosystems). The PCR reactions were performed in triplicate. After an initial activation step at 50°C for 2 min and a hot start at 95°C for 10 min, PCR cycles consisted of 40 cycles at 95°C for 15 sec and 60°C for 60 sec. Dissociation curve analysis were performed for each reaction to ensure amplification of specific products.

Genes and primers are shown in table 1. Gene expression was normalized with the mean of 18S mRNA content and calculated relative to controls or contralateral kidneys. Results were finally expressed as 2–ΔCT (CT threshold cycle), which is an index of the relative amount of mRNA expression in each tissue.

**Table 1:** qRT-PCR primers.

Primers:	Forward:	Reverse:	Amplicon length (bp):
18S	AGTCCCTGCCCTTTGTACACAC	AACCATCCAATCGGTAGTAGCG	51
TNF-α	GGCTGCCTTGGTTCAGATGT	CAGGTGGGAGCAACCTACAGTT	63
IL-6	AGACAAAGCCACCACCCCTAA	CTCGTTCTGTGACTGCAGCTTATC	69
MCP-1	ACTTGGGCACATTGCTTTCCT	TTTTGTGTTCACCATCCTTGCA	84
ICAM-1	GGCTGTGCACTGCAACAAGA	TGTGGCAATGCCAAATCCT	75
NA-K ATPase	AGAGGAAGATCGTGGAGTTCACC	AATTCCTCCGGGTCTTGCAG	75
AQP-1	GAAGGCTGGATTCTATCTACATAAGTCC	TTGTCTAGCTGAAACCGTGGG	86
AQP-2	CTGTGGAGCTTTTCCTGACC	TAGTGGATCCCGAGAAGGTG	100
AQP-3	CTCATGGTGGTTTCCTCACC	CAAGGATACCCAGGGTGACA	24

### Immunohistochemistry

Three-µm-thick sections were cut from paraffin embedded kidney tissue. For evaluation of renal morphology sections were stained by Periodic Acid-Schiff (PAS). For immunohistochemical aquaporin-2 (AQP-2) staining sections were deparaffinized and endogenous peroxidase was blocked by incubation with 3.5% H_2_O_2_ for 30 minutes. Antigens were retrieved by boiling sections for 15 minutes in Tris/EGTA buffer (pH 9.0). Endogenous biotin was blocked using a ready-to-use blocking kit (X0590, DAKO, Glostrup, Denmark). Unspecific binding of free aldehyde groups was blocked by 30 minutes incubation in 50 mM NH_4_Cl followed by blocking of unspecific antibody binding by 30 minutes incubation in 1%BSA, 0,2% gelatin and 0,05% saponin. The primary antibody (Anti-AQP2, 1∶500, 7661AP) was incubated overnight followed by incubation with a secondary peroxidase-conjungated goat-anti-rabbit antibody (1∶300, DAKO, Glostrup, Denmark). Sensitivity was increased and background was reduced by the ready-to-use ABC Kit (PK 4000, Vectastain, Vector). Then the peroxidase activity was visualized by ten minutes incubation in 3.3-diaminobenzidine tetrachloride. Subsequently the sections were counterstained with hematoxylin.

Finally, the sections were scanned using APERIO scanscope (Aperio, Vista, United States). The intensity of the immunohistochemical staining of each section was quantified using APERIO image scope software.

### Urinary markers of acute kidney injury

Neutrophil gelatinase-associated lipocalin (NGAL) was measured using a NGAL ELISA kit (Bioporto diagnostics A/S, Gentofte, Denmark). N-acetyl-b-D-glucosaminidase (NAG) was measured by a modified enzyme assay at pH 4.25 using p-nitrophenyl-N-acetyl-b-D-glucosaminide as substrate (Sigma-Aldrich, Zwijndrecht, the Netherlands). The activity of alanine aminopeptidase (AAP) was detected with the modified enzymatic assay using alanine-p-nitroanilide (Sigma-Aldrich, Zwijndrecht, the Netherlands).

### Statistical analyses

Grouped and paired data was analyzed using the repeated measurement ANOVA and is presented as mean±standard error of the mean (SEM). Data on specific times has been analyzed using Wilcoxon signed rank test or Man Whitney U test depending on paired or non-paired data and is presented as mean±interquartile range. P<0.05 was considered significant.

## Results

### Effect on clinical parameters

**Figure 2:**
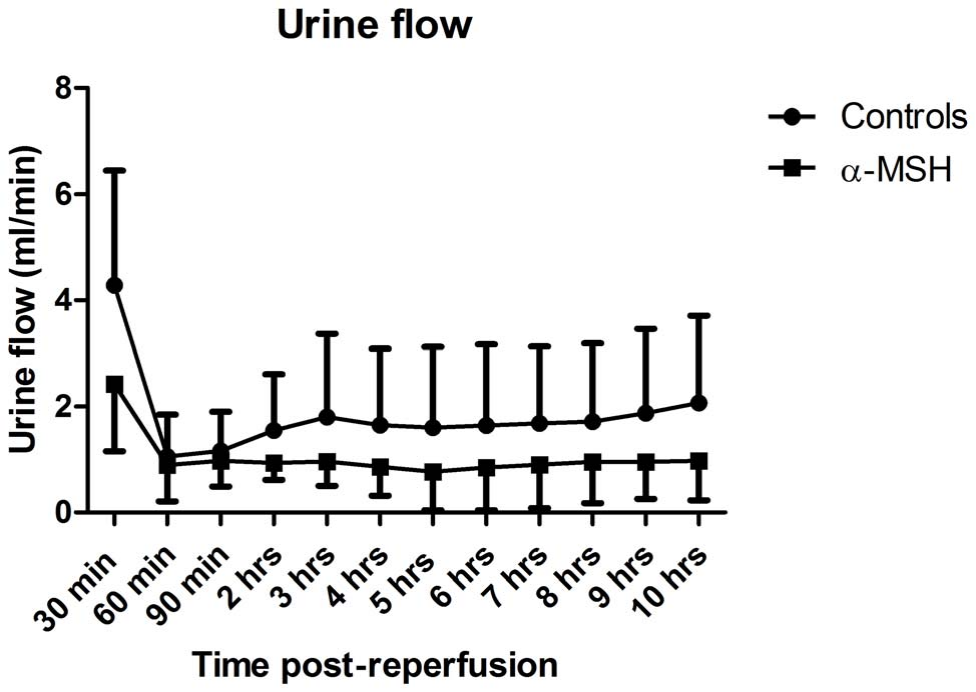
Effect of α-MSH on urine flow. Urine flow was significantly lower in the α-MSH group compared to controls during ten hours follow-up.

**Figure 3:**
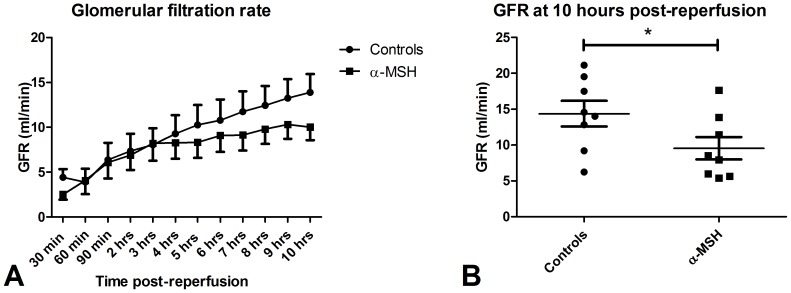
Effect of α-MSH on GFR. Figure 3A shows the recovery of renal function during follow-up of ten hours. At ten hours post-reperfusion α-MSH significantly reduced GFR (Figure 3B; * = p<0.05).

**Figure 4:**
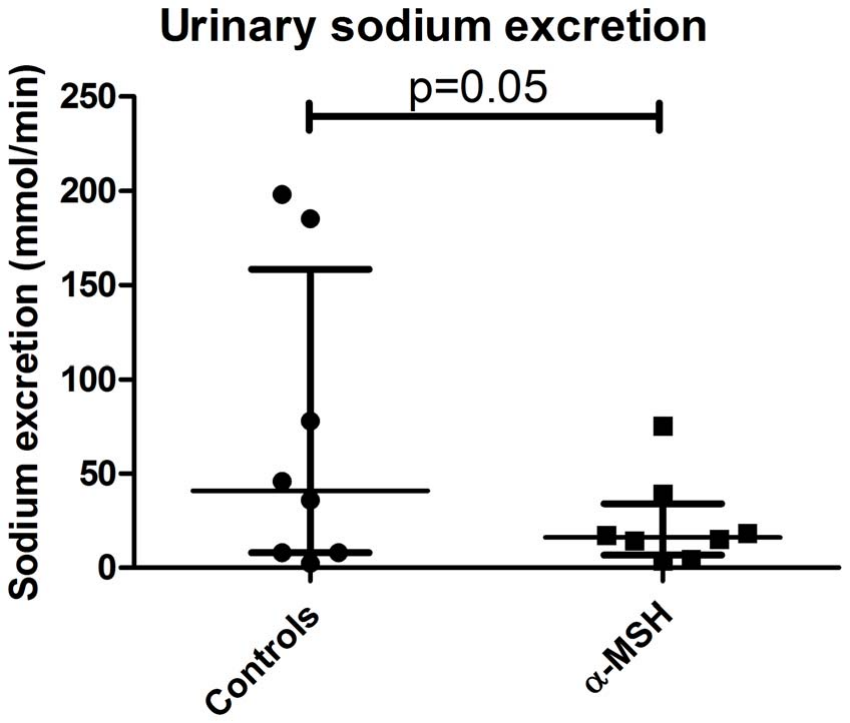
Effect of α-MSH on Sodium excretion. Sodium excretion per minute was reduced by α-MSH (* = p<0.05).

During ten hours follow-up, urine output was significantly lower in the α-MSH group compared to the controls (Figure 2, p<0.05). None of the controls or α-MSH treated animals were anuric. α-MSH treatment tended to impair recovery of GFR (Figure 3A) during ten hours follow-up compared to controls. At ten hours post-reperfusion, GFR of α-MSH treated animals was significantly lower compared to controls (Figure 3B; 8.2±5.7−13.3 ml/min vs. 14.3±10.1−19 ml/min; p<0.05). Sodium excretion was also reduced by α-MSH treatment at ten hours post-reperfusion compared to controls (Figure 4; 2.8±1.2−4.5 mmol/min vs. 5.4±4.2−5.8 mmol/min; p<0.05). Plasma creatinine and urea levels increased during follow-up in both groups, but did not differ significantly (Figure S1A and S1B).

**Figure 5:**
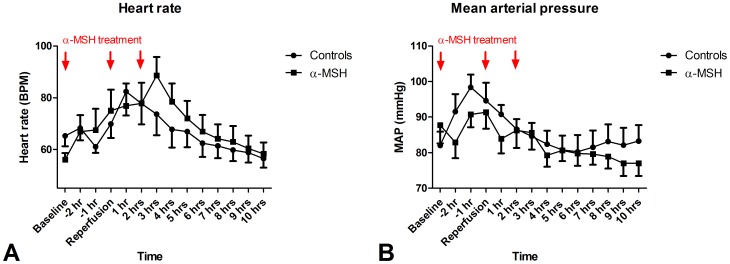
Effect of α-MSH on hemodynamic parameters. α-MSH tended to increase heart rate (Figure 5A) and reduce MAP (Figure 5B) after each dose.

**Figure 6:**
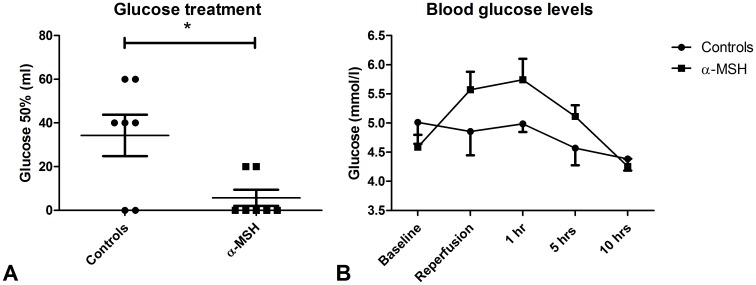
Effect of α-MSH on glucose levels. α-MSH significantly reduced the need of glucose treatment (Figure 6A; * = p<0.05) and tended to prevent a decrease of blood glucose levels (Figure 6B).

During surgery, heart rate of all animals increased. After reperfusion, heart rate decreased to baseline levels in both groups. Each dose of α-MSH tended to increase heart rate by 10 beats per minute (BPM) (Figure 5A). Mean arterial pressure (MAP) increased during surgery. After reperfusion, MAP decreased to baseline levels during the ten hour follow-up. Each dose of α-MSH tended to decrease MAP by approximately 10 mmHg. After the first two doses this appeared within the first hour after administration and after the third dose this was observed in the second hour after administration (Figure 5B). One α-MSH treated animal required a bolus of one liter Ringer's acetate and adrenalin (0.025 mg) five minutes post-reperfusion because of rapidly decreasing MAP to levels below 35 mmHg. The recipient stabilized immediately after one dose of adrenalin and one liter Ringer's acetate. None of the control animals required adrenalin treatment. During ten hours follow-up one α-MSH treated animal required two boluses of one liter Ringer's acetate to maintain MAP above 60 mmHg while in none of the controls MAP dropped below 60 mmHg. The need for glucose supplementation to maintain blood glucose levels above 4.0 mmol/l was significantly lower in α-MSH treated animals (Figure 6A; 0±0−20 ml 50% glucose vs. 40±0−60 50% glucose; p<0.05). Despite more glucose supplementation, blood glucose levels of controls tended to be lower compared to α-MSH treated animals (Figure 6B). One control- and one α-MSH treated recipient transplanted simultaneously were excluded from these analyses because of an excessive need for glucose supplementation, 420 and 200 ml 50% glucose, respectively.

**Figure 7:**
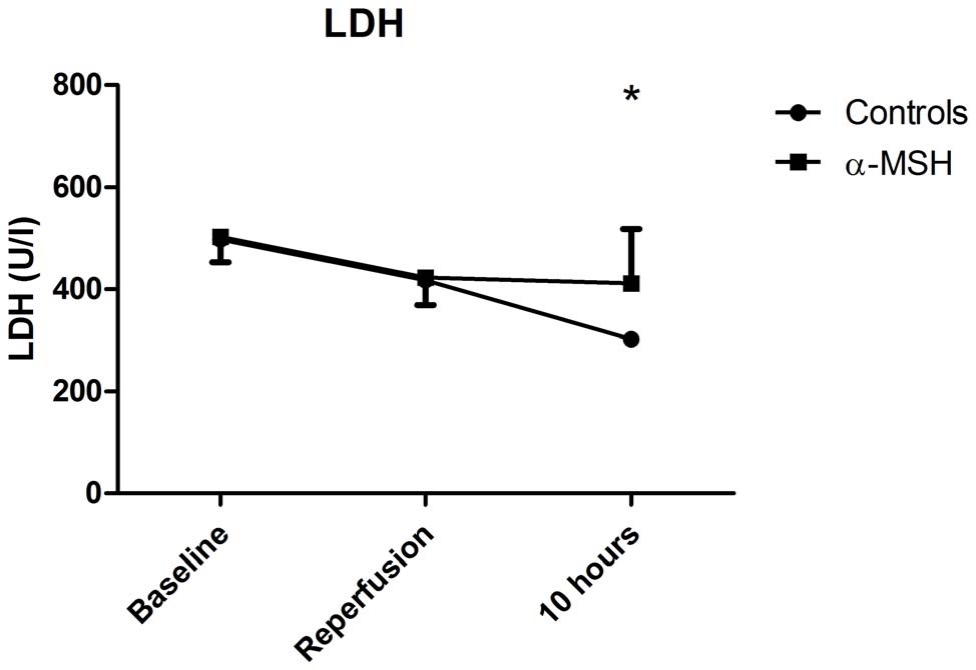
Effect of α-MSH on LDH. At ten hours post-reperfusion LDH was higher in α-MSH treated animals compared to controls (Figure 7; * = p<0.05).

Hemoglobin levels decreased during surgery and ten hours follow-up. α-MSH treatment did not affect hemoglobin levels compared to controls (Figure S2A). No differences in blood pH or lactate levels were observed between α-MSH treatment and controls (Figure S2B and S2C). α-MSH treatment did not influence ASAT or ALAT levels (Figure S3A and S3B). LDH levels decreased in the control group during follow-up, while such change was not seen in the α-MSH group at ten hours post-reperfusion (Figure 7, p<0.05).

### Effect on urinary markers of acute kidney injury

**Figure 8:**
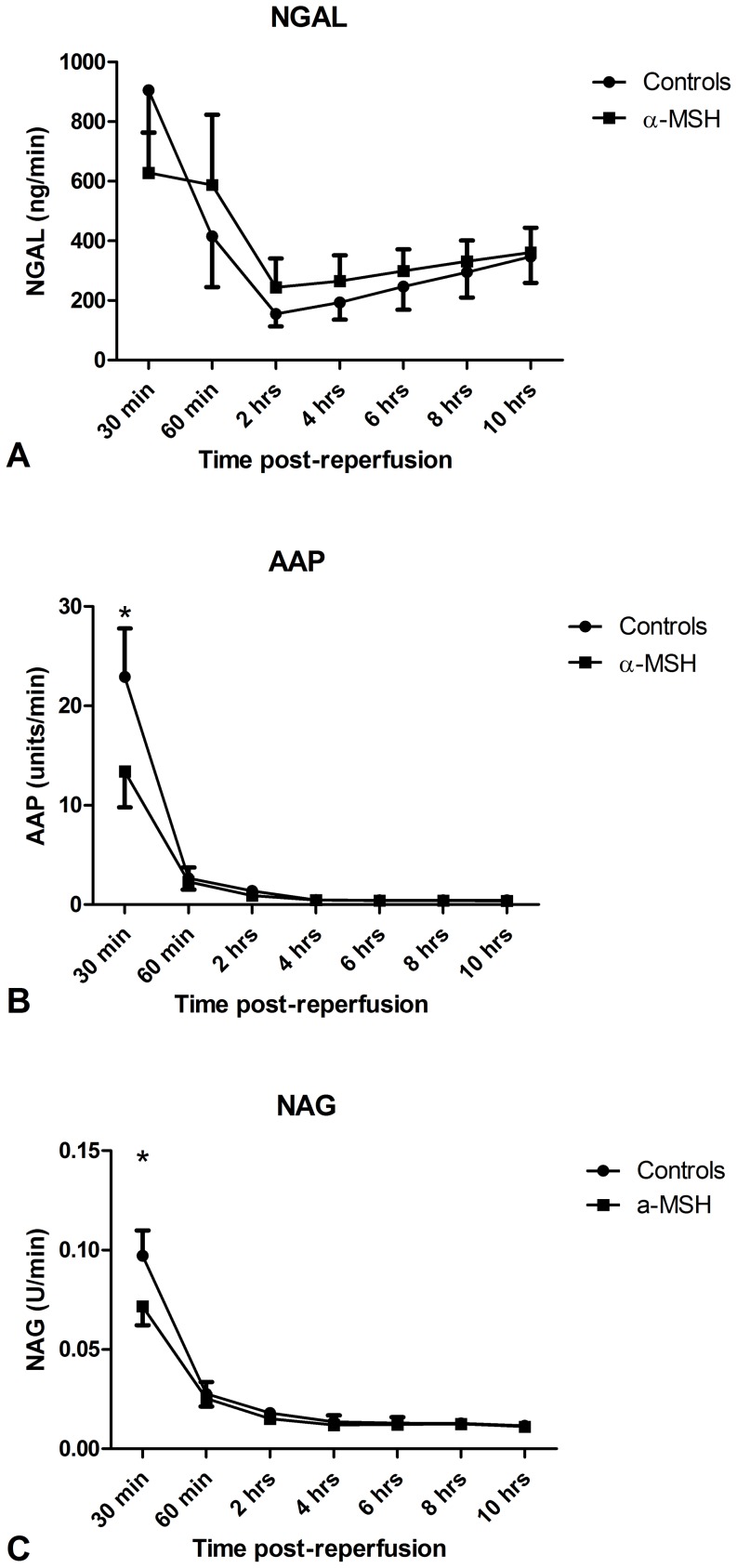
Effect of α-MSH on urinary markers of acute renal injury. α-MSH significantly reduced AAP and NAG excretion in the first thirty minutes (Figure 8B and 8C; * = p<0.05).

Urinary excretion of renal injury markers, respectively NGAL, NAG and AAP, was highest in the first 30 minutes post-reperfusion. NGAL excretion did not differ between α-MSH treated animals and controls (Figure 8A). However, α-MSH treatment significantly reduced AAP and NAG excretion rate during follow-up. The difference is based on a significant reduction in the first thirty minutes (Figure 8B and 8C; p<0.05).

### Effect on inflammation

**Figure 9:**
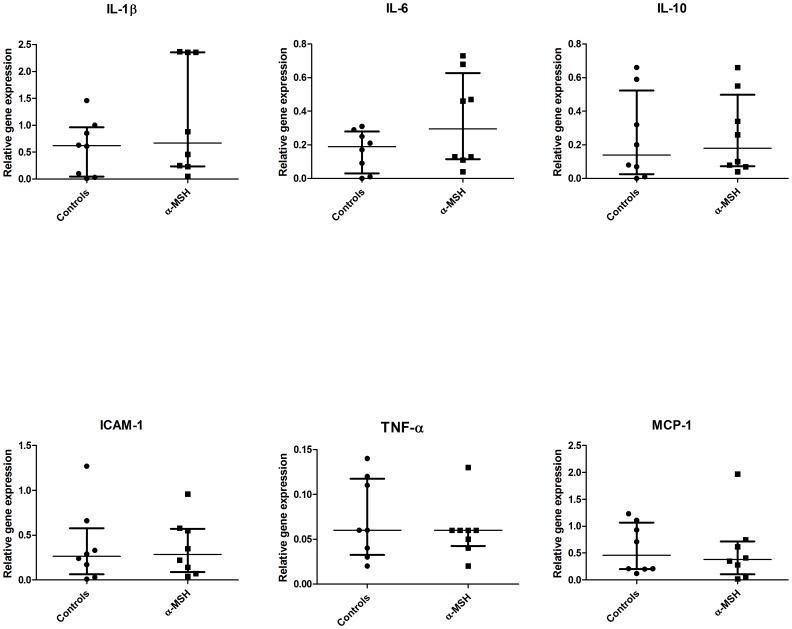
Effect of α-MSH inflammatory markers. α-MSH treatment did not significantly affect mRNA expression of IL-1β, IL-6, IL-10, ICAM-1, TNF-α or MCP-1.

The effect of α-MSH on inflammatory markers was measured in cortical tissue collected at ten hours post-reperfusion (Figure 9). Expression of interleukin-1β (IL-1β), -6 (IL-6) or -10 (IL-10) was not significantly affected by α-MSH treatment. No differences were observed in mRNA levels of intercellular adhesion molecule-1 (ICAM-1), tumour necrosis factor-α (TNF-α) or monocyte chemoattractant protein-1 (MCP-1).

### Effect on renal morphology and aquaporins

**Figure 10:**
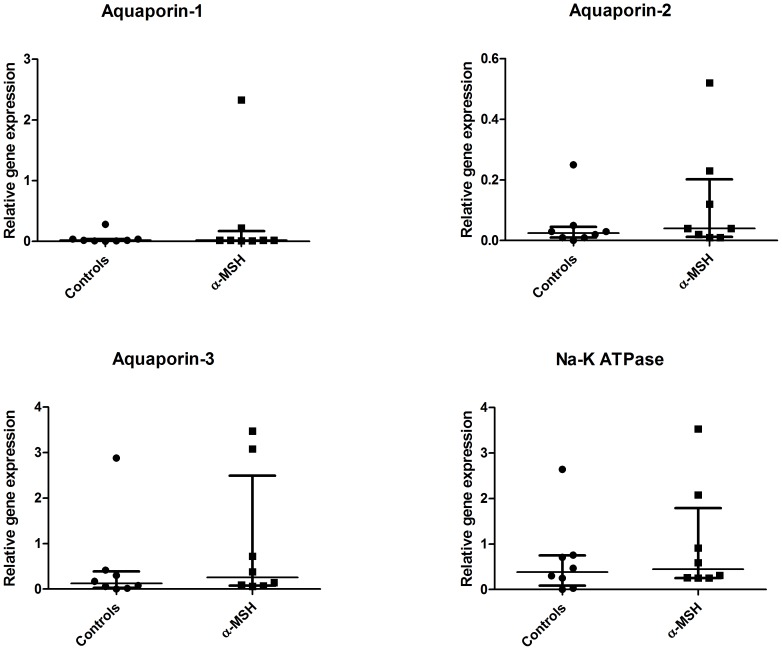
The effect of α-MSH on aquaporins and Na-K ATPase. No significant differences were observed in mRNA expression of aquaporines or Na-K ATPase mRNA expression between controls and α-MSH treated animals at ten hours post-reperfusion.

**Figure 11:**
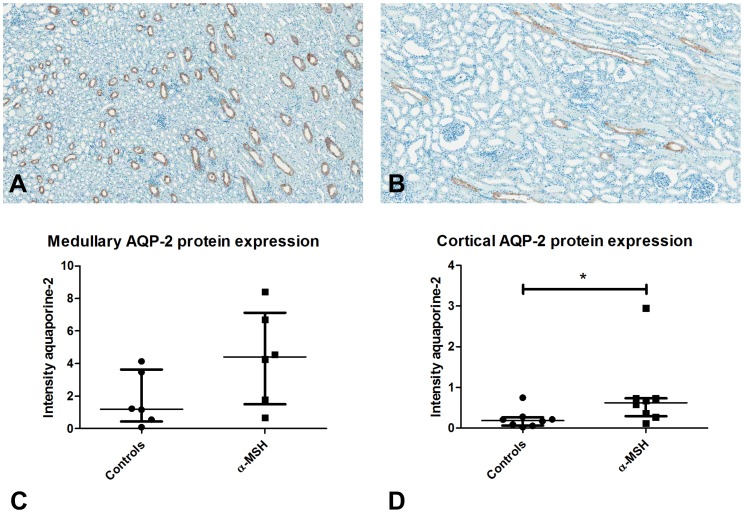
The effect of α-MSH on AQP-2 protein expression. Figure 11A and 11B show sections of medullary and cortical AQP-2 staining, respectively. No differences were observed in medullary AQP-2 expression (C), while α-MSH significantly increased cortical protein expression of AQP-2 (D; * = p<0.05).

No differences were observed in renal morphology between α-MSH treated animals and controls. In addition to morphological changes, we investigated the effect of α-MSH on renal expression of aquaporins and Na-K ATPase. At ten hours post-reperfusion, cortical mRNA expression levels of aquaporin-1 (AQP-1), -2 (AQP-2), -3 (AQP-3) and Na-K ATPase were not significantly affected by α-MSH treatment (Figure 10). However, quantification of immunohistochemical staining showed increased cortical AQP-2 protein expression following α-MSH treatment indicative of increased AQP-2 activity in the renal cortex (Figure 11 A-D; 0.6±0.3−0.7 vs. 0.2±0.1−0.3; p<0.05).

## Discussion

This study was designed to test the hypothesis that α-MSH treatment of recipients of a DBD donor kidney improves early graft function and reduces inflammation. Surprisingly, α-MSH treatment impaired recovery of GFR following DBD transplantation. Furthermore, no anti-inflammatory capacities of α-MSH were observed after ten hours follow-up. Urinary excretion of markers of acute kidney injury, AAP and NAG, were reduced by α-MSH treatment, but only in the first 30 minutes post-reperfusion and urinary NGAL excretion was not affected. Besides, α-MSH administration was associated with a slightly increased AQP-2 protein expression in renal cortex. However, it is uncertain whether these small changes in urinary markers of acute kidney injury and aquaporin expression are clinically relevant.

α-MSH impaired GFR recovery after DBD kidney transplantation. This may be explained by changes in hemodynamics following each dose of α-MSH. Heart rate increased by approximately ten beats per minute in the hour following α-MSH administration, while α-MSH administration was associated with 10 mmHg reduction of MAP. In the literature, the effects of α-MSH on hemodynamics are variable. In particular the hemodynamic effect of intravenous administration of α-MSH is debatable and mainly based on studies in rodents [20]–[23]. However, in sheep α-MSH (75 µg/kg) decreased MAP, while heart rate and aortic flow increased directly following treatment suggesting a decrease of systemic vascular resistance. Blood flow to lungs and heart increased, but renal blood flow was not significantly affected [24].

In this study, 200 µg/kg α-MSH was used, which was chosen based on the previously shown protective effect against renal I/R injury [16], [17]. Gong et al. demonstrated the protective effect of α-MSH in renal I/R injury in rats [16]. Furthermore, Simmons et al. tested the protective effects of 200 µg/kg AP214, which is a synthetic analogue of α-MSH with 6 lysine residues added to its amino terminus, in renal I/R in pigs [17]. However, in both the rodent- and porcine I/R model the effect of α-MSH or AP214 on hemodynamics was not investigated. It was therefore unexpected that α-MSH seemed to directly affect hemodynamics. Possibly, the hemodynamic changes counteracted the proposed renoprotective effect of α-MSH.

Recently, it has been shown that α-MSH is able to induce vasodilatation by endothelial derived nitric oxide (NO) via activation of melanocyte receptor-1 (MCR1) [25]. Based on this mechanism one could speculate that α-MSH might increase GFR due to the expression of MCR1 in renal glomeruli [26]. However, we did not observe this in our model. A potential stimulative effect of α-MSH on GFR might be counteracted by systemic vasodilatation and subsequent decreased MAP. As a consequence, this would lead to production of catecholamines and activation of the renin angiotensin aldosterone system, activation of which would result in increased sodium retention and renal vasoconstriction [27]. Our results indeed show reduced sodium excretion, however we acknowledge the precise hemodynamic effects of α-MSH still have to be unraveled including the possible differential effects on afferent and efferent glomerular arterioles. We speculate the hemodynamic changes observed as a consequence of α-MSH administration caused impaired GFR in this model.

The follow-up of ten hours after transplantation is relatively short, which is a limitation. Based on the protective effect of α-MSH against acute kidney injury, the short follow-up was chosen specifically to study the effects of α-MSH on immediate graft function and acute inflammation following DBD transplantation [12]–[17]. The effect of α-MSH on long-term renal function after transplantation and structural injury was not investigated and cannot be deducted from our present results. However, α-MSH impaired recovery of GFR and failed to show any reduction of inflammatory qRT-PCR markers. We believe it is rather unlikely that the short follow-up period masked the capacity of α-MSH to improve early graft function following DBD transplantation.

α-MSH can bind to four different melanocortin receptors (MCR1, MCR3, MCR4, MCR5). These different receptors are responsible for pleiotropic function of α-MSH, like regulation of pigmentation, hemodynamics, glucose metabolism and immunomodulation [28], [29]. The anti-inflammatory effect is mainly mediated by MCR1, but also by MCR3 and MCR4. The anti-inflammatory capacities of α-MSH are based on modulation of antigen presenting cells and reduced production of cytokines such as IL-1β, TNF-α and IL-6 [28], [30], [31]. Although MCR1 and MCR3 expression in the kidney is slightly decreased after renal I/R, renal expression of these receptors suggests α-MSH treatment might be locally protective [28], [32]. Chiao et al. showed using isolated perfused kidneys after renal ischemia that addition of α-MSH to the perfusion solution improves renal function indicating that α-MSH mediated renoprotection is based on a renal binding to MCR's [13]. In our study, no differences in cortical expression of several markers of inflammation was found between controls and α-MSH treated animals. The local anti-inflammatory effects, as shown in models of acute kidney injury [10], [12], [15], might be counteracted by systemic effects of MCR1,3–5. The hemodynamic effects and the effect on blood glucose levels suggest systemic activity of α-MSH in this porcine transplantation model. Selective agonists of MCR1, MCR3 or MCR4 might induce anti-inflammatory effects without possible adverse effects of activating the complete MCR system.

Renal I/R injury results in down-regulation of aquaporins and sodium transporters [29], [33]. Aquaporins and sodium transporters are important for the renal reabsorptive capacity of water and sodium. In models of acute kidney injury, prevention of down-regulation of aquaporins and sodium transporters is associated with reduced sodium and water excretion and improved renal function [11], [14], [16], [34]. In this study α-MSH seemed to slightly increased cortical AQP-2 protein expression. Clinically, urine flow was halved and sodium excretion reduced, but recovery of GFR was impaired. These functional renal effects are presumably caused by changes in hemodynamics and not by the small changes in AQP-2 expression.

The anti-inflammatory effects and improvement of renal function observed after α-MSH treatment in various models of acute renal injury suggested α-MSH could prevent DGF and PNF following DBD kidney transplantation. However, α-MSH seemed to reduce MAP and impaired short-term recovery of renal function. Anti-inflammatory capacities of α-MSH were not observed. In conclusion, α-MSH treatment of recipients did not improve early graft function or reduce acute inflammation in this short-term model of deceased donor kidney transplantation.

## Supporting Information

Figure S1 **Effect of α-MSH on plasma creatinine and urea levels.**
(TIF)

Figure S2 **Effect of α-MSH on plasma hemoglobin, pH and lactate levels.**
(TIF)

Figure S3 **Effect of α-MSH on plasma ASAT and ALAT levels.**
(TIF)
