# Supplementary figures and images for: α-Melanocyte Stimulating Hormone Treatment in Pigs Does Not Improve Early Graft Function in Kidney Transplants from Brain Dead Donors
Source: PLoS One. 2014 Apr 11;9(4):e94609. doi: 10.1371/journal.pone.0094609 (PMC3984270; doi:10.1371/journal.pone.0094609)

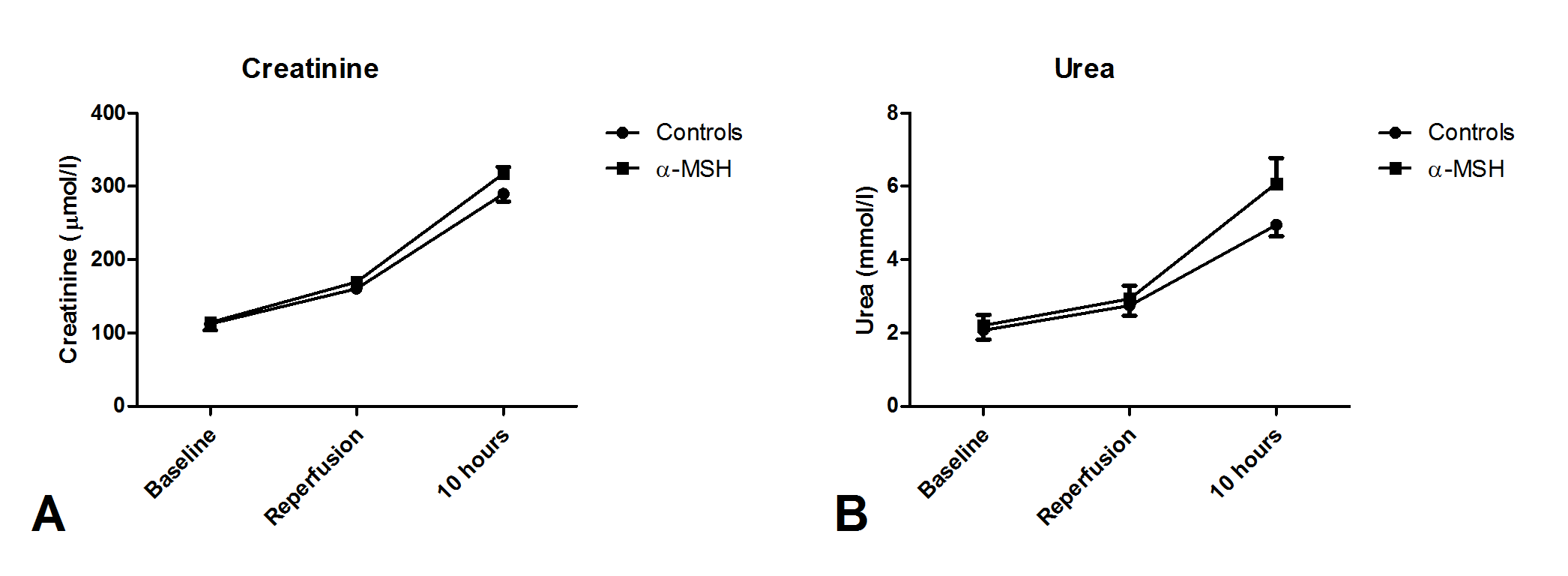

Supplement: Figure S1 — Effect of α-MSH on plasma creatinine and urea levels. (TIF) [file pone.0094609.s001.tif]

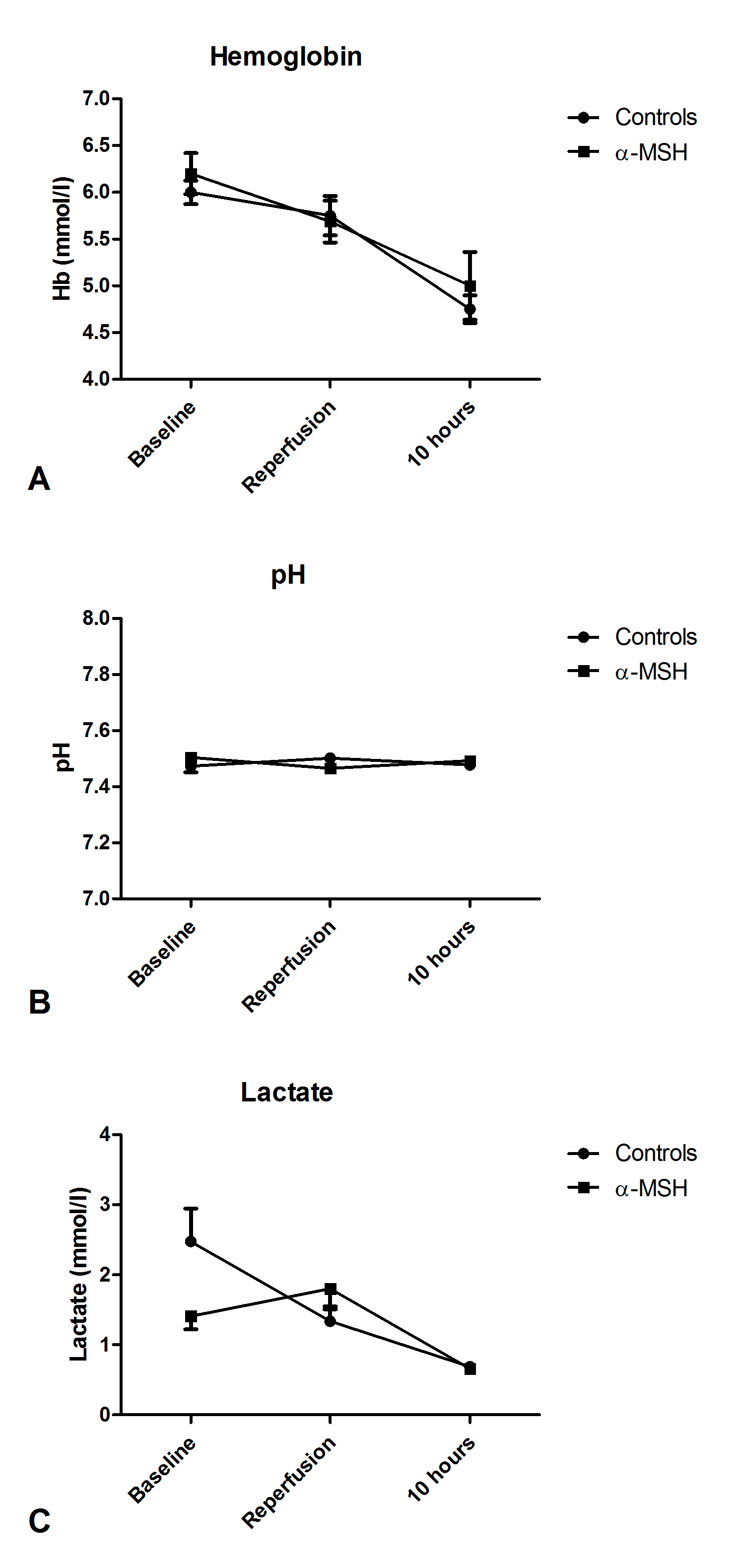

Supplement: Figure S2 — Effect of α-MSH on plasma hemoglobin, pH and lactate levels. (TIF) [file pone.0094609.s002.tif]

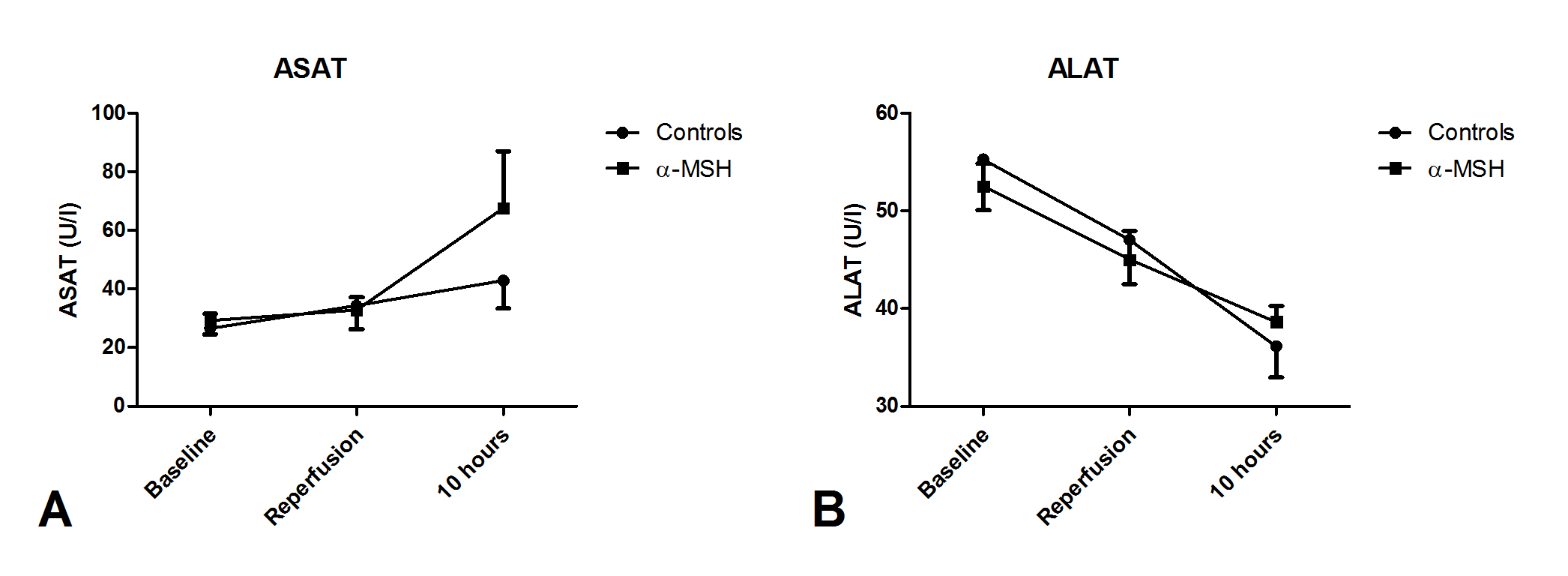

Supplement: Figure S3 — Effect of α-MSH on plasma ASAT and ALAT levels. (TIF) [file pone.0094609.s003.tif]
